# Elevated parathyroid hormone one year after kidney transplantation is an independent risk factor for graft loss even without hypercalcemia

**DOI:** 10.1186/s12882-022-02840-5

**Published:** 2022-06-17

**Authors:** Manabu Okada, Yoshihiro Tominaga, Tetsuhiko Sato, Toshihide Tomosugi, Kenta Futamura, Takahisa Hiramitsu, Toshihiro Ichimori, Norihiko Goto, Shunji Narumi, Takaaki Kobayashi, Kazuharu Uchida, Yoshihiko Watarai

**Affiliations:** 1 Department of Transplant and Endocrine Surgery, Japanese Red Cross Nagoya Daini Hospital, 2-9 Myoken-cho, Showa-ku, Nagoya, Aichi 4668650 Japan; 2 Department of Diabetes and Endocrinology, Japanese Red Cross Nagoya Daini Hospital, Showa-ku, Nagoya, Aichi Japan; 3 Department of Renal Transplant Surgery, Aichi Medical University School of Medicine, Nagakute, Aichi Japan; 4 Department of Renal Transplant Surgery, Masuko Memorial Hospital, Nakamura-ku, Nagoya, Aichi Japan

**Keywords:** Hyperparathyroidism, Multivariate analysis, Normocalcemia, Kidney transplantation

## Abstract

**Background:**

Hypercalcemic hyperparathyroidism has been associated with poor outcomes after kidney transplantation (KTx). However, the clinical implications of normocalcemic hyperparathyroidism after KTx are unclear. This retrospective cohort study attempted to identify these implications.

**Methods:**

Normocalcemic recipients who underwent KTx between 2000 and 2016 without a history of parathyroidectomy were included in the study. Those who lost their graft within 1 year posttransplant were excluded. Normocalcemia was defined as total serum calcium levels of 8.5–10.5 mg/dL, while hyperparathyroidism was defined as when intact parathyroid hormone levels exceeded 80 pg/mL. The patients were divided into two groups based on the presence of hyperparathyroidism 1 year after KTx. The primary outcome was the risk of graft loss.

**Results:**

Among the 892 consecutive patients, 493 did not have hyperparathyroidism (HPT-free group), and 399 had normocalcemic hyperparathyroidism (NC-HPT group). Ninety-five patients lost their grafts. Death-censored graft survival after KTx was significantly lower in the NC-HPT group than in the HPT-free group (96.7% vs. 99.6% after 5 years, respectively, *P* < 0.001). Cox hazard analysis revealed that normocalcemic hyperparathyroidism was an independent risk factor for graft loss (*P* = 0.002; hazard ratio, 1.94; 95% confidence interval, 1.27–2.98).

**Conclusions:**

Normocalcemic hyperparathyroidism 1 year after KTx was an independent risk factor for death-censored graft loss. Early intervention of elevated parathyroid hormone levels may lead to better graft outcomes, even without overt hypercalcemia.

**Supplementary Information:**

The online version contains supplementary material available at 10.1186/s12882-022-02840-5.

## Background

Secondary hyperparathyroidism is a frequent complication of chronic kidney disease (CKD) and increases the risk of mortality and various other complications [1]. Although successful kidney transplantation (KTx) can alleviate secondary hyperparathyroidism to some extent [2, 3], hyperparathyroidism often persists and adversely affects clinical outcomes despite improved kidney function [2–6]. Elevated parathyroid hormone (PTH) levels promote bone resorption, calcium (Ca) reabsorption from the tubular tubes, and Ca absorption from the intestinal tract by increasing the production of 1,25-dihydroxycholecalciferol, often causing hypercalcemia [7]. Concerning the management of hyperparathyroidism after KTx, persistent hypercalcemia has been internationally recognized as the most common therapeutic indication [8–11], as hypercalcemia has been associated with poor outcomes. Egbuna et al. indicated in a retrospective study of 422 kidney transplant patients that hypercalcemia adversely affected mortality and graft prognosis [10]. Moore et al. also demonstrated the mortality risk of hypercalcemia in their study of 303 kidney transplant patients [11]. However, a high level of PTH alone is not generally a factor in making therapeutic decisions because it is not known whether high PTH levels without hypercalcemia may adversely affect kidney-graft function after KTx [12]. Therefore, this retrospective cohort study of 892 kidney transplant patients aimed to identify the impact of elevated PTH levels without hypercalcemia on kidney-graft outcomes. To the best of our knowledge, there have been no reports focusing on the clinical implications of high PTH levels without hypercalcemia after KTx.

## Methods

### Study design and subjects

Consecutive patients who underwent KTx between January 2000 and December 2016 at the Japanese Red Cross Nagoya Daini Hospital (Nagoya, Japan) were included in the study. Data were collected on December 31, 2020. Our patient-exclusion criteria were as follows: those for whom data were lacking; those who had hypercalcemia or hypocalcemia within 1 year of KTx; those who were undergoing treatment with calcimimetics; those who had lost their kidney graft within 1 year of KTx; or those who were under 16 years of age at KTx. Furthermore, those who underwent parathyroidectomy (PTx) were also excluded because their PTH values were affected by the autografted parathyroid function. Hypercalcemia was defined as total serum Ca levels > 10.5 mg/dL. Hypocalcemia was defined as total serum Ca levels < 8.5 mg/dL. Normocalcemic hyperparathyroidism was defined as intact PTH levels of > 80 pg/mL without hypo/hypercalcemia 1 year after KTx. Intact PTH level has been reported to be ≤80 pg/mL in over 98% of healthy individuals irrespective of vitamin D status in both PTH assays used in this study [13].

Patients who met the inclusion criteria were divided into two groups based on the presence (or absence) of hyperparathyroidism: the HPT-free group, comprising patients without hyperparathyroidism and the NC-HPT group, comprising patients with normocalcemic hyperparathyroidism 1 year after successful KTx. Each patients’ sex, age, body mass index (BMI), dialysis vintage, number of HLA mismatches, positivity of donor-specific HLA antibody (DSA), laboratory data, mean blood pressure (MBP) 1 year after KTx, and graft survival were documented. The primary outcome was the risk of death-censored graft loss. Blood sample analyses were performed on all patients every month for 1 year following transplantation and every second month thereafter. The values of serum Ca and intact PTH obtained from blood samples 1 year after KTx were used for patient enrollment and classification. This study is reported according to Strengthening The Reporting of Observational studies in Epidemiology guidelines.

### Measurements

Serum Ca and phosphorus (P) levels were measured using standard methods. Intact PTH levels were measured using second-generation immunoassays: an electrochemical luminescence immunoassay (SRL, Tokyo, Japan, www.srl-group.co.jp, reference range [10–65 pg/mL]), or an enzyme immunoassay (TOSOH Company, Tokyo, Japan, www.tosoh.co.jp, reference range [9–80 pg/mL]). When serum albumin values were < 4.0 g/dL, all serum Ca values were corrected with serum albumin values as follows [14].

$$\mathrm{Corrected}\ \mathrm{Ca}\ \left(\mathrm{mg}/\mathrm{dL}\right)=\mathrm{measured}\ \mathrm{total}\ \mathrm{Ca}\ \left(\mathrm{mg}/\mathrm{dL}\right)+0.8\ast \left(4.0-\mathrm{serum}\ \mathrm{albumin}\ \left[\mathrm{g}/\mathrm{dL}\right]\right).$$

The estimated glomerular filtration rate (eGFR) was evaluated using the creatinine equation provided by the Japanese Society of Nephrology [15].

### Immunosuppression

According to protocols conducted at the Japanese Red Cross Nagoya Daini Hospital, the main immunosuppressive regimens were calcineurin inhibitors (cyclosporine or tacrolimus), mycophenolic acid, mizoribine, everolimus, and glucocorticoids. Basiliximab was used as induction therapy. Furthermore, rituximab administration or splenectomy was used as induction therapy in anti-donor antibody-positive patients with KTx, except in those with low antibody titers.

### Statistical analysis

Pearson’s chi-squared test was used to analyze nominal variables, and the Mann–Whitney U test was used for continuous variables. All results are presented as median (interquartile range) because of their non-normal distribution, confirmed by the Kolmogorov–Smirnov test. Spearman’s rank correlation coefficient was used to evaluate the correlations among the variables (Electronic Supplementary Material (ESM 1)). Kaplan-Meier survival curves and logrank tests were used to estimate the graft survival rates. The Cox proportional hazards regression was performed to evaluate the risk of death-censored graft loss. Donor age [16], BMI [17], diabetes mellitus [18], preformed DSA [19], ABO blood type incompatibility [20], serum P [21], and eGFR [22] 1 year post KTx were included as covariates in the multivariate analysis; these factors have been reported as renal prognostic factors after KTx in previous studies [16–22]. To reduce selection bias and potential confounding effects, propensity score (PS)-based methods were also employed. A logistic regression model involving 11 covariates was used to derive PSs. These covariates included eight continuous variables (recipient age, BMI, dialysis vintage, donor age, serum P, serum Ca, eGFR, and MBP 1 year post KTx) and three nominal variables (ABO blood type incompatibility, diabetes mellitus, and preformed DSA). Inverse probability of treatment weighting for NC-HPT was used to estimate the hazard ratio (HR) for death-censored graft loss by the Cox proportional hazards model (ESM 2). In addition, PS matching in a 1:1 raio between the NC-HPT and HPT-free groups was also performed to confirm the robustness of the results of other analyses (ESM 3, ESM 4). The crude and multivariable-adjusted risk for death-censored graft loss with intact PTH levels categorized by quartiles were also examined. SPSS version 23.0 (IBM Corp., Armonk, NY, USA) and EZR version 1.40 [23] were used for statistical analyses. Statistical significance was set at *P* < 0.05.

## Results

### Patient baseline characteristics

**Fig. 1:**
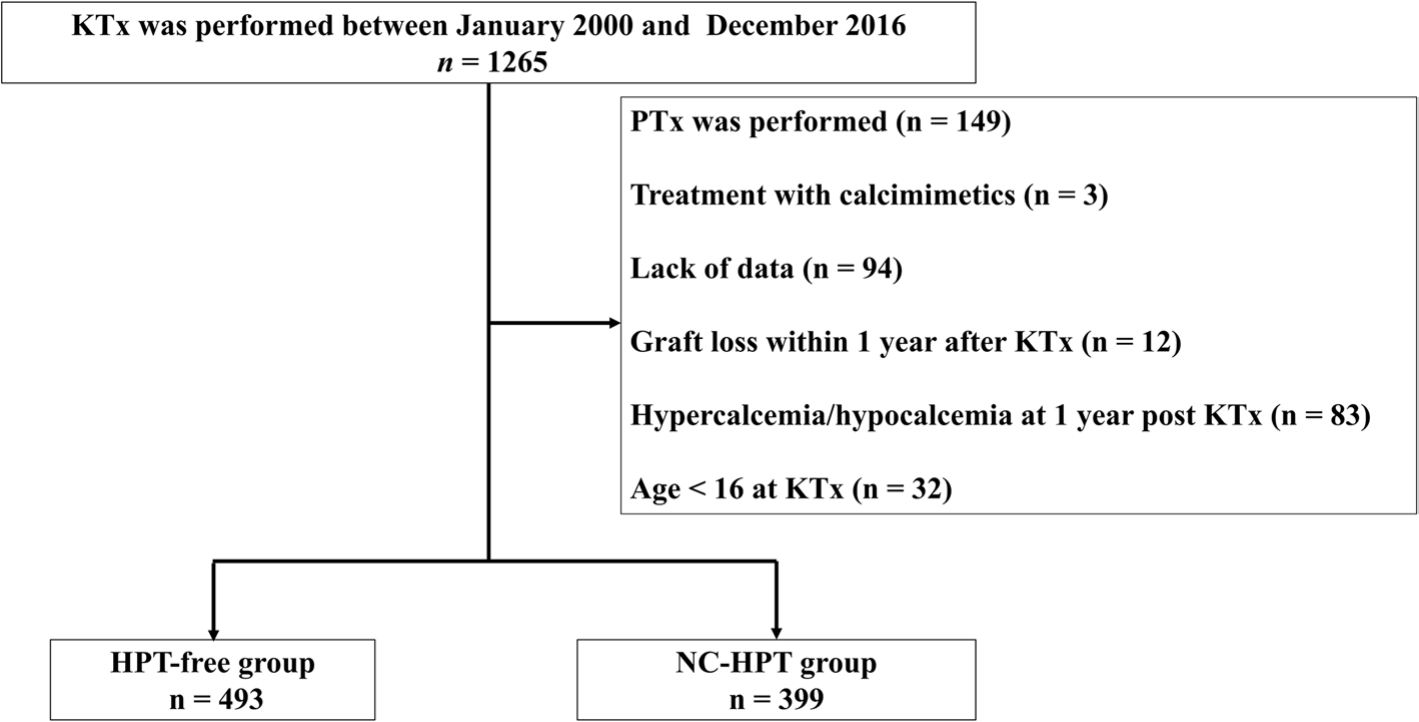
Study selection flowchart. *HPT*, hyperparathyroidism; *NC-HPT*, normocalcemic hyperparathyroidism; *PTx*, parathyroidectomy; *KTx*, kidney transplantation

**Fig. 2:**
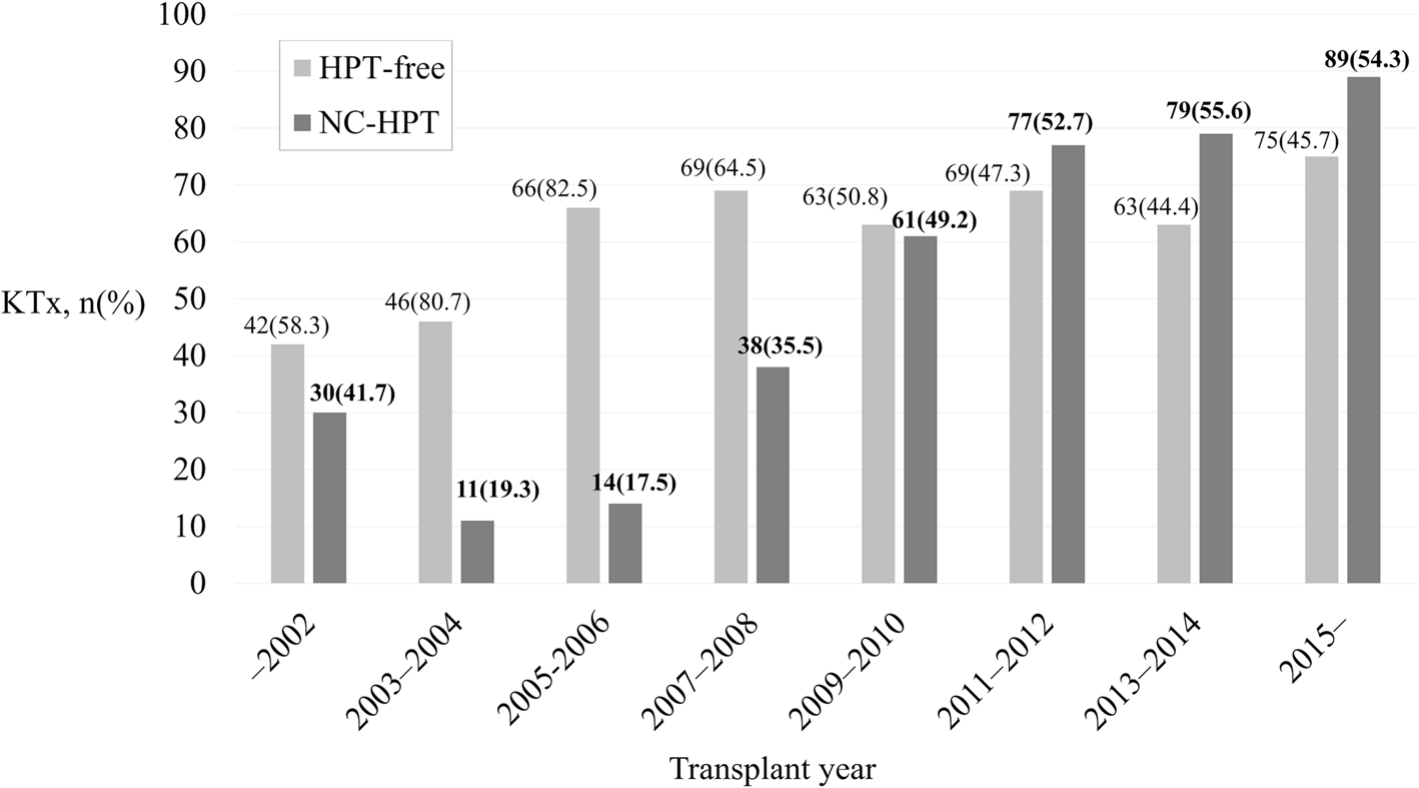
The HPT-free and NC-HPT group by transplant years. *HPT*, hyperparathyroidism; *NC-HPT*, normocalcemic hyperparathyroidism; *PTx*; parathyroidectomy; *KTx*, kidney transplantation

A total of 892 patients met the inclusion criteria for the study (median observation period: 129 [interquartile range, 93–174] months). Of the 892 patients, 493 were assigned to the HPT-free group, and 399 were assigned to the NC-HPT group (Fig. 1). The number and proportion of patients in the NC-HPT group tended to increase over the years (Fig. 2). The intact PTH levels of the NC-HPT group were consistently higher than those of the HPT-free group from the time of KTx to 1 year after KTx (ESM 5). Patient baseline characteristics are presented in Table 1. There were significant differences between the HPT-free and the NC-HPT groups in recipient age, donor age, BMI, dialysis vintage, serum Ca, serum P, intact PTH, eGFR and MBP 1 year after KTx. Other characteristics did not differ between the two groups (Table 1). Although no patients were treated with calcimimetics during the follow-up period, oral vitamin D supplementation was introduced in 9.9% (49/493) of patients in the HPT-free group 97 (65–133) months after KTx, and 11.3% (45/399) of patients in the NC-HPT group 82 (45–114) months after KTx, mainly for the treatment of osteoporosis (*P* = 0.591). There was a weak correlation between intact PTH and age, dialysis vintage, BMI, serum Ca, serum P, and eGFR, with an absolute correlation coefficient of less than 0.2 (ESM 1).

**Table 1:** Patient characteristics and clinical outcomes

	HPT-free *N* = 493	NC-HPT *N* = 399	*P*-value
Baseline characteristics			
Recipient age (years)	43 (31–57)	45 (36–59)	0.012*
Recipient sex (male, %)	321 (65.1)	251 (62.9)	0.540
Body mass index (kg/m^2^)	21.3 (19.4–23.6)	22.3 (19.8–25.0)	0.001*
Dialysis vintage (months)	4 (0–21)	7 (0–38)	0.009*
Preemptive KTx (%)	190 (38.5)	144 (36.1)	0.495
Diabetes mellitus (%)	104 (21.1)	87 (21.8)	0.861
Living/deceased donor (%)	483 (98.0)/10 (2.0)	386 (96.7)/13 (3.3)	0.347
Donor age (years)	57 (50–62)	60 (51–65)	< 0 .001*
Donor sex (male, %)	181 (36.8)	134 (33.6)	0.355
HLA AB mismatch	2 (1–3)	2 (1–3)	0.234
HLA DR mismatch	1 (1–2)	1 (1–2)	0.293
Preformed DSA (%)	28 (5.8)	21 (5.3)	0.902
ABO blood type incompatible KTx (%)	132 (26.8)	124 (31.1)	0.181
Lab data one year post KTx			
Corrected calcium (mg/dL)	9.8 (9.6–10.0)	9.7 (9.4–10.0)	< 0.001*
Phosphorus (mg/dL)	3.4 (3.0–3.7)	3.3 (2.9–3.6)	0.013*
Intact PTH (pg/mL)	55.0 (43.0–66.0)	104.0 (91.0–136.0)	< 0.001*
eGFR (mL/min/1.73m^2^)	46.2 (39.2–53.6)	42.1 (34.7–50.4)	< 0.001*
MBP one year post KTx (mmHg)	90.7 (83.0–98.3)	92.7 (84.0–100.0)	0.032*
Clinical outcomes			
Death (%)	28 (5.7)	18 (4.5)	0.527
Death-censored graft loss (%)	42 (8.5)	53 (13.3)	0.022*
Chronic allograft nephropathy	10 (2.0)	24 (6.0)	0.002*
Chronic rejection	18 (3.7)	12 (3.0)	0.596
Vascular complication	4 (0.8)	4 (1.0)	0.763
Recurrent nephritis	2 (0.4)	4 (1.0)	0.278
Infection	2 (0.4)	1 (0.3)	0.691
Others	4 (0.8)	7 (1.8)	0.204
Unknown	2 (0.4)	1 (0.3)	0.691
Follow-up period (months)	146 (100–189)	116 (86–147)	< 0.001*

Data for continuous variables are presented as median (interquartile range)

*DSA* Donor-specific HLA antibody, *eGFR* Estimated glomerular filtration rate, *HPT* Hyperparathyroidism, *MBP* Mean blood pressure, *NC-HPT* Normocalcemic hyperparathyroidism, *PTH* Parathyroid hormone, *KTx* Kidney transplantation

*P-value < 0.05

The dialysis vintage was defined as 0 when preemptive KTx was performed

### Graft survival

**Fig. 3:**
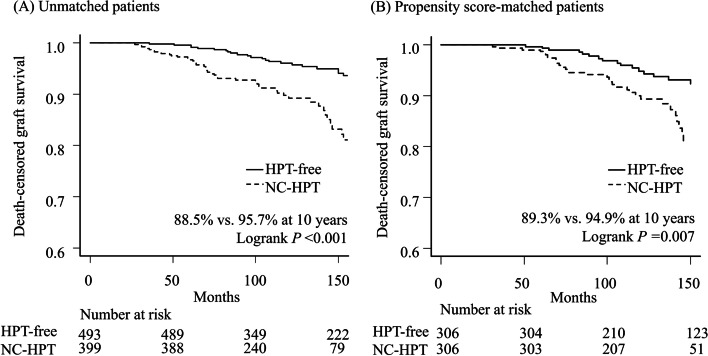
Death-censored graft survival curves according to status of hyperparathyroidism. *HPT*, hyperparathyroidism; *NC-HPT*, normocalcemic hyperparathyroidism

Graft loss was observed in 95 patients, with a more frequent occurrence in the NC-HPT group than in the HPT-free group (13.3% vs 8.5%, respectively, *P* = 0.022) despite the shorter follow-up period in the NC-HPT group (116 months vs 146 months, respectively, *P* < 0.001) (Table 1). The death-censored graft survival in the NC-HPT group was significantly lower than that in the HPT-free group (96.7% vs. 99.6% at 5 years and 88.5% vs 95.7% at 10 years, *P* < 0.001) (Fig. 3A). Even after PS matching of 306 recipients from each group, death-censored graft survival of NC-HPT recipients was still inferior to that of HPT-free recipients (98.7% vs 99.5% at 5 years, and 89.3% vs 94.9% at 10 years, *P* = 0.007) (Fig. 3B). The proportion of graft loss due to chronic allograft nephropathy was significantly higher in the NC-HPT group than in the HPT-free group (5.8% vs. 1.9%, *P* = 0.002) (Table 1).

### Risk of death-censored graft loss

**Fig. 4:**
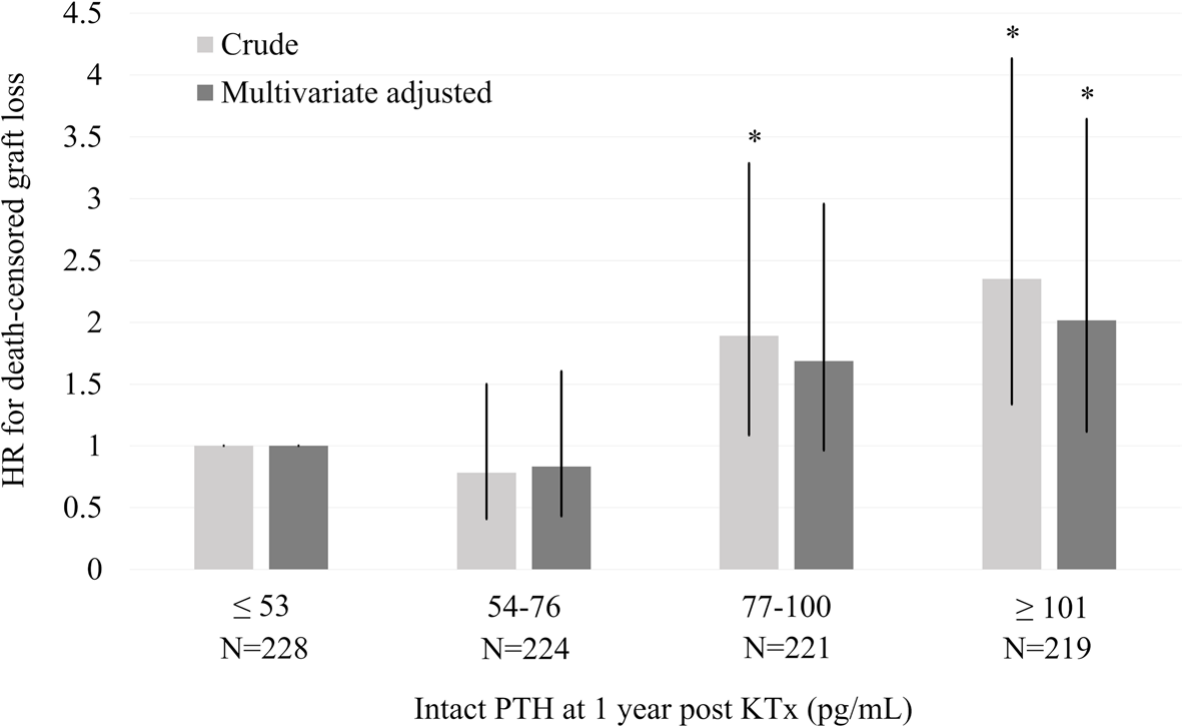
HRs for graft loss according to categories of PTH using Cox proportional hazard model. The multivariable-adjusted analysis included donor age, body mass index, diabetes mellitus, preformed donor-specific HLA antibody, ABO blood type incompatible KTx, phosphorus, and eGFR at one year post-KTx. *P < 0.05. *eGFR*, estimated glomerular filtration rate; *HR*, hazard ratio; *PTH*, parathyroid hormone; *KTx*, kidney transplantation

The univariate Cox proportional hazards revealed that the independent risk factors for death-censored graft loss were donor age (*P* = 0.020; HR, 1.025; 95% confidence interval [CI], 1.004–1.048), BMI (*P* = 0.005; HR, 1.075; 95% CI, 1.022–1.130), diabetes mellitus (*P* = 0.028; HR, 1.719; 95% CI, 1.061–2.785), DSA positivity (*P* = 0.003; HR, 3.117; 95% CI, 1.485–6.546), serum P 1 year post-KTx (*P* < 0.001; HR, 1.705; 95% CI, 1.298–2.241), eGFR 1 year post-KTx (*P* < 0.001; HR, 0.953; 95% CI, 0.935–0.972), and normocalcemic hyperparathyroidism (*P* < 0.001; HR, 2.290; 95% CI, 1.518–3.457) (Table 2). The multivariate Cox proportional hazards model confirmed a significantly increased risk of death-censored graft loss in normocalcemic hyperparathyroidism (*P* = 0.002; HR, 1.944; 95% CI, 1.268–2.980) (Table 2). In addition, the Cox proportional hazards model adjusted by PS-based methods also revealed significantly higher risk of death-censored graft loss in the NC-HPT group than in the HPT-free group (Table 3). Figure 4 shows crude (unadjusted) and multivariable-adjusted HRs for death-censored graft loss with categories of intact PTH levels at 1 year after KTx. There was a trend to an increase in the multivariate-adjusted HR of death-censored graft loss with intact PTH levels of 77–100 pg/mL (*P* = 0.067, HR, 1.688; 95% CI: 0.963–2.958) and > 100 pg/mL (*P* = 0.020, HR, 2.017; 95% CI: 1.117–3.644) (Fig. 4).

**Table 2:** Risk factors for death-censored graft loss

	Univariate analysis			Multivariate analysis		
	*P*-value	HR	95% CI	*P*-value	HR	95% CI
Male recipient	0.129	1.403	0.906–2.173			
Male donor	0.397	0.829	0.538–1.279			
Recipient age (years)	0.349	0.993	0.978–1.008			
Donor age (years)	0.020*	1.025	1.004–1.048	0.550	1.007	0.983–1.032
Body mass index (kg/m^2^)	0.005*	1.075	1.022–1.130	0.034*	1.060	1.004–1.119
Dialysis vintage (months)	0.459	1.002	0.997–1.006			
Preemptive KTx	0.399	0.817	0.511–1.307			
Diabetes mellitus	0.028	1.719	1.061–2.785	0.514	1.192	0.704–2.018
Deceased donor KTx	0.843	1.123	0.355–3.552			
HLA AB mismatch	0.733	0.966	0.792–1.179			
HLA DR mismatch	0.910	0.981	0.701–1.372			
Preformed DSA	0.003*	3.117	1.485–6.546	0.007*	2.834	1.331–6.034
ABO blood type Incompatible KTx	0.240	1.322	0.830–2.106	0.722	1.089	0.682–1.738
Corrected calcium at one year post KTx (mg/dL)	0.542	0.840	0.480–1.472			
Phosphorus at one year post KTx (mg/dL)	< 0.001*	1.705	1.298–2.241	0.009*	1.501	1.106–2.038
eGFR at one year post KTx (mL/min/1.73m^2^)	< 0.001*	0.953	0.935–0.972	0.004*	0.971	0.951–0.991
NC-HPT	< 0.001*	2.290	1.518–3.457	0.002*	1.944	1.268–2.980

*DSA* Donor-specific HLA antibody, *eGFR* Estimated glemerular filtration rate, *HPT* Hyperparathyroidism, *NC-HPT* Normocalcemic hyperparathyroidism, *PTH* Parathyroid hormone, *KTx* Kidney transplantation

* *P*-value < 0.05

**Table 3:** Propensity-score adjusted cox regression analysis for death-censored graft loss of the NC-HPT group with reference to the HPT-free group

Method	*P*-value	HR	95% CI
IPTW (*N* = 821)	< 0.001*	2.421	1.560–3.758
Propensity score matching (*N* = 612)	0.008*	1.978	1.197–3.269

*IPTW* Inverse probability of treatment weighting, *HPT* Hyperparathyroidism, *NC-HPT* Normocalcemic hyperparathyroidism

* *P*-value < 0.05

## Discussion

The multivariate analyses in this study demonstrated that BMI, DSA, serum P, and eGFR 1 year post-KTx were risk factors for graft loss, which is consistent with previous reports [17, 19, 21, 22]. Even with adjustment for these risk factors, normocalcemic hyperparathyroidism 1 year after KTx was shown to be an independent risk factor for death-censored graft loss. In addition, the multivariable-adjusted HRs of graft loss with category levels of PTH showed that graft prognosis could become worse as PTH values increase. The increasing trend of normocalcemic hyperparathyroidism shown in this study may be due to an increase of elderly patients or a drastic decrease of pretransplant PTx accompanied by the recent developments of medical treatments [24]. Taken together with its increasing tendency, normocalcemic hyperparathyroidism after KTx could be a non-negligible disease entity. Our results are consistent with other studies indicating that PTH may be a risk factor for graft loss [4, 25]. These results suggest the need for active management of elevated PTH levels after KTx, even in the absence of hypercalcemia. Furthermore, for the prevention of post-KTx hyperparathyroidism, active intervention for elevated PTH prior to KTx should be considered.

Since GFR reduction is a direct cause of PTH elevation, it is difficult to determine whether high PTH after KTx is mainly due to low GFR or to parathyroid gland hyperplasia [26]. In addition, hyperparathyroidism is also associated with hypertension [27]. Therefore, although adjusted by mulitivariate analysis and PS-based methods in this study, the inferior graft survival in the NC-HPT group may have been influenced by low GFR or hypertension. However, there are several possible mechanisms by which elevated PTH levels can worsen graft outcomes. Normocalcemic hyperparathyroidism in non-CKD patients reportedly increases nephrolithiasis [28–31] and cardiovascular risk [32–34]. Furthermore, some observational studies in cases of secondary hyperparathyroidism have demonstrated the association of PTH with renal anemia [35] or immunodeficiency [36]. In addition, it has been recently reported that PTH increases energy consumption by transforming adipocytes to brown adipocytes, leading to cachexia-like pathology seen in patients with malignancy [37]. More importantly, PTH can induce various effects by promoting the secretion of fibroblast growth factor 23 (FGF23) [38]. FGF23 is a humoral factor produced by osteocytes and has been recognized as a significant predictor of life prognosis in patients undergoing dialysis [39], in whom it can induce cardiac hypertrophy [40], renal anemia [41], immunodeficiency [42], and chronic inflammation [43]. Additionally, both PTH and FGF23 increase pathological fibrosis in CKD [44], a condition known to adversely affect kidney grafts. The various pathologies mentioned above may explain the association between normocalcemic hyperparathyroidism and inferior graft outcome in the present study.

The optimal management of normocalcemic hyperparathyroidism in kidney transplant patients is controversial due to a lack of consensus. However, aggressive treatment for elevated PTH after KTx may improve clinical outcomes, as several reports have demonstrated that therapeutic intervention for normocalcemic hyperparathyroidism in non-CKD patients can reduce complication risks, including bone lesions [45, 46], nephrolithiasis [47, 48], cardiovascular disease [34], and reduced quality of life [49].

Although the “Kidney Disease: Improving Global Outcomes” guidelines recommend active vitamin D and bisphosphonates as medical treatments for hyperparathyroidism in the first year after KTx, these treatments are often difficult to implement because of concerns about hypercalcemia, insufficient kidney function, and/or low bone turnover [50]. Therefore, interventions and therapeutic options for hyperparathyroidism with normocalcemia should be tailored to individual cases in actual clinical practice. PTx or medical treatments such as calcimimetics, vitamin D, or bisphosphonate should be prescribed according to underlying CKD conditions, including levels of Ca and PTH, degree of enlargement of parathyroid glands, kidney function, bone loss, and cardiovascular risk in the patient.

This study has several limitations, most of which are due to its retrospective nature, its confinement to a single center, the inherent possibility of unmeasured confounders, selection bias, and absence of data regarding endogenic vitamin D levels, FGF23, other bone biomarkers, and renal lithiasis. Hence, further studies with larger sample sizes are necessary to validate the findings of this study.

## Conclusions

Hyperparathyroidism 1 year after KTx was found to be an independent risk factor for death-censored graft loss, even without overt hypercalcemia. Early management and intervention of elevated PTH levels may contribute to better graft outcomes after kidney transplantation.

## Supplementary Information

**Additional file 1.**

**Additional file 2.**

**Additional file 3.**

**Additional file 4.**

**Additional file 5.**

**Additional file 6.**

## Data Availability

The datasets generated and/or analyzed during the current study are available from the corresponding author on reasonable request.

## Abbreviations

BMI: Body mass index
Ca: Calcium
CI: Confidence interval
CKD: Chronic kidney disease
DSA: Donor-specific HLA antibody
eGFR: Estimated glomerular filtration rate
FGF23: Fibroblast growth factor 23
HR: Hazard ratio
IPTW: Inverse probability of treatment weighting
KTx: Kidney transplantation
MBP: Mean blood pressure
P: Phosphorus
PTH: Parathyroid hormone
PS: Propensity score
PTx: Parathyroidectomy
