# Supplementary figures and images for: Elevated parathyroid hormone one year after kidney transplantation is an independent risk factor for graft loss even without hypercalcemia
Source: BMC Nephrol. 2022 Jun 17;23:212. doi: 10.1186/s12882-022-02840-5 (PMC9205154; doi:10.1186/s12882-022-02840-5)

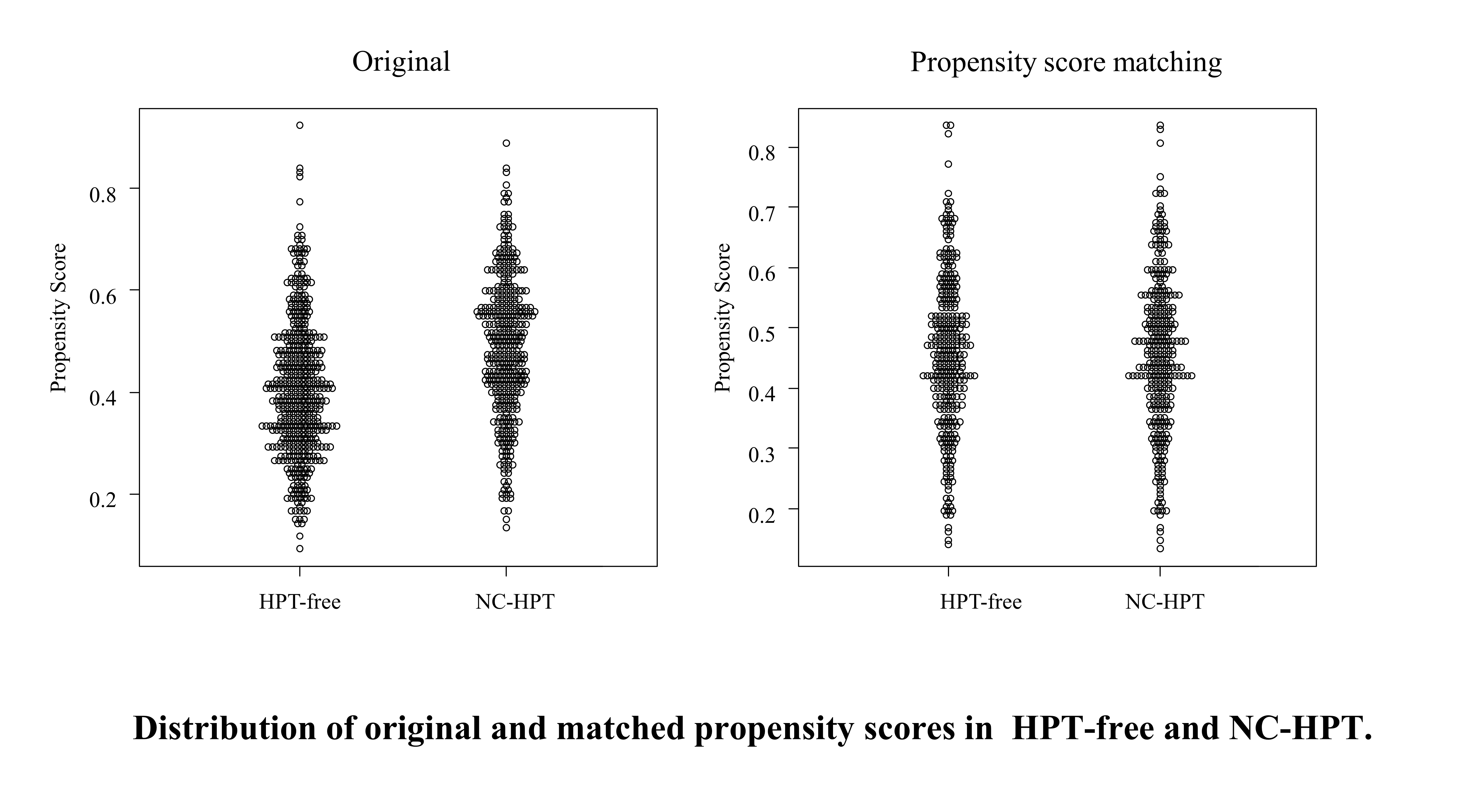

Supplement: Supplementary file 4 — Additional file 4. [file 12882_2022_2840_MOESM4_ESM.tif]

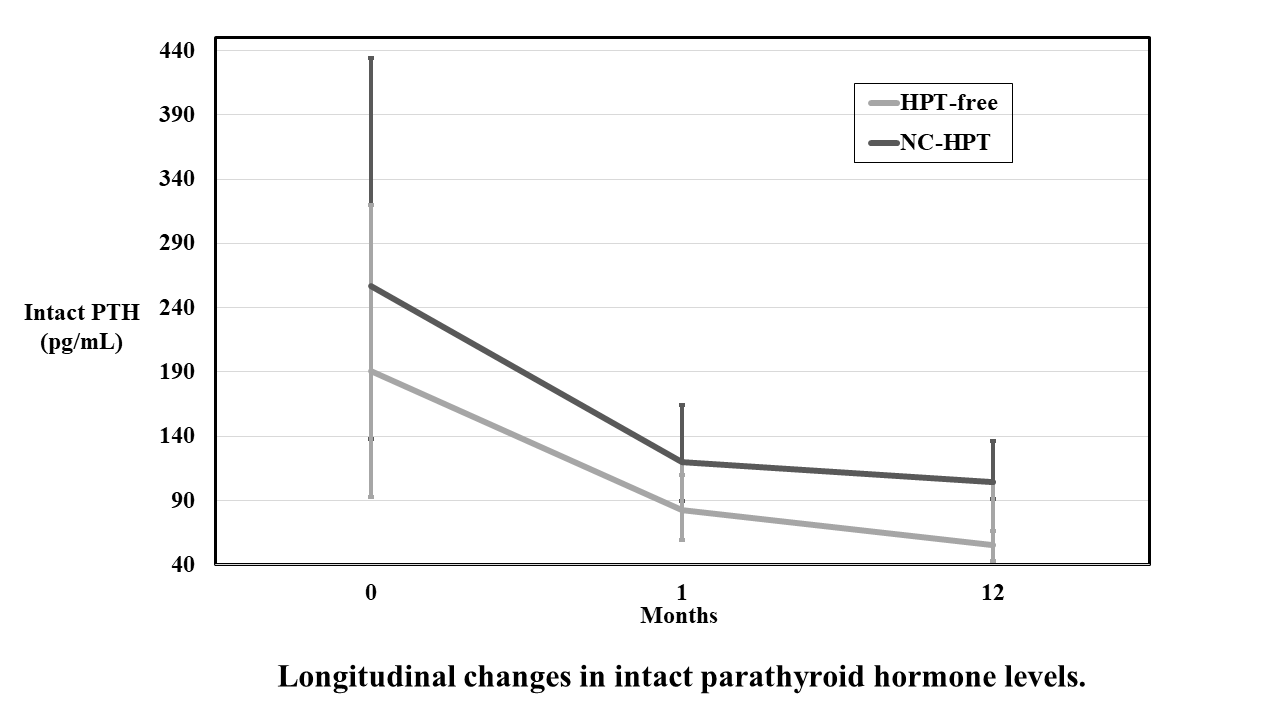

Supplement: Supplementary file 5 — Additional file 5. [file 12882_2022_2840_MOESM5_ESM.tif]
